# Grayscale median (GSM) post-processing, posterizing, and color mapping for carotid ultrasound

**DOI:** 10.1590/1677-5449.202200811

**Published:** 2023-02-10

**Authors:** Pedro Luciano Mellucci, Matheus Bertanha, Rodrigo Gibin Jaldin, Winston Bonetti Yoshida, Marcone Lima Sobreira

**Affiliations:** 1 Universidade Estadual Paulista - UNESP, Botucatu, SP, Brasil.

**Keywords:** carotid artery diseases, carotid stenosis, Doppler ultrasound, afterimage, computer-assisted image interpretation

## Abstract

Factors related to atherosclerotic plaques may indicate instability, such as ulcerations, intraplaque hemorrhages, lipid core, thin or irregular fibrous cap, and inflammation. The grayscale median (GSM) value is one of the most widespread methods of studying atherosclerotic plaques and it is therefore important to comprehensively standardize image post-processing. Post-processing was performed using Photoshop 23.1.1.202. Images were standardized by adjusting the grayscale histogram curves, setting the darkest point of the vascular lumen (blood) to zero and the distal adventitia to 190. Posterization and color mapping were performed. A methodology that presents the current state of the art in an accessible and illustrative way should contribute to the dissemination of GSM analysis. This article describes and illustrates the process step by step.

## INTRODUCTION

Randomized studies conducted between 1990 and 2000 were able to identify risk of stroke in symptomatic and asymptomatic patients with extracranial carotid disease based on the percentage of stenosis observed in the internal carotid, encompassing a considerable number of patients and establishing guidelines that are employed worldwide for carotid endarterectomy or angioplasty based on the degree of stenosis.^1^^-^ 5 The main pathophysiologic mechanisms underlying cerebral ischemic events are caused by embolization of atherosclerotic debris or thrombi originating from unstable plaques, primarily located in the carotid bifurcations.^6^

However, cerebral hypoperfusion is not the principal pathophysiologic mechanism of strokes of extracranial carotid origin. In these cases, the principal factor causing stroke is displacement of atherosclerotic debris and thrombi originating in unstable plaques.^6^

Use of percentage stenosis as the only indicator for surgery has been shown to be insufficient and there is a debate about adding new markers related to plaque characteristics, such as ulcerations, lipid core volume, presence of intraplaque hemorrhage, fibrous cap thickness, and inflammation, reinforcing the concept that an unstable plaque is the determinant factor for indicating surgery, rather than taking stenosis as the only predictor. Many different factors can be associated with instability of carotid plaques, giving rise to a wide field of research attempting to define predictors of ischemic cerebral events that could replace use of percentage stenosis alone, such as ulcerations, intraplaque hemorrhages, lipid core, thin or irregular fibrous cap, and inflammation.^7^^,^ 8

One of the most widely studied factors related to the atherosclerotic plaque is its grayscale median (GSM) value. A study published in 2010 by the Asymptomatic Carotid Stenosis and Risk of Stroke (ACSRS) study group reported that GSM values lower than 30 were associated with an increased risk of atherosclerotic embolic events, with a significant increase in relative risk at values below 15.^7^

While study of GSM has been widely reported in the literature, it is not routinely performed by most sonographers, and its use is restricted to a few teaching and research centers, possibly because the majority of ultrasound machines do not offer software for automatic GSM assessment. There is also a considerable degree of variation in the information available on methods for measuring GSM, ranging from use of built-in software,^9^ i.e., programs installed in the ultrasound equipment - to post-processing - which consists of analyzing echographic images on a computer.

In order to contribute to dissemination of the technique and to improve reproducibility, it is important to standardize post-analysis in a manner that is clear and comprehensible to sonographers who have had technical training in post-processing of images.

## MATERIALS AND METHODS

### Image acquisition

Ultrasound images were obtained in B-mode by a single sonographer with qualifications from the Brazilian College of Radiology and Diagnostic Imaging (CBR) and the Brazilian Society of Angiology and Vascular Surgery (SBACV), using a single Logiq S8 machine (General Electric, Boston, Massachusetts, United States) with a multifrequency linear transducer (8.5-11 MHz) set to 10 MHz. Focal distance was defined at the posterior tunica adventitia, the time gain compensation (TGC) levers were set to the center, and all gain parameters were standardized at the machine’s original presets, including 69 dB dynamic range. All images were acquired longitudinally.

The images obtained comprised 1,552 x 970 pixels, containing a rectangular region of interest comprising 934 x 840 pixels, without the titles, configuration buttons, time, date, and grayscale bar. We chose to perform post-processing on the entire image (1,552 x 970 pixels), to avoid undesired exclusions and to maintain the grayscale bar.

### Post-processing: standardization of the GSM

**Figure 1:**
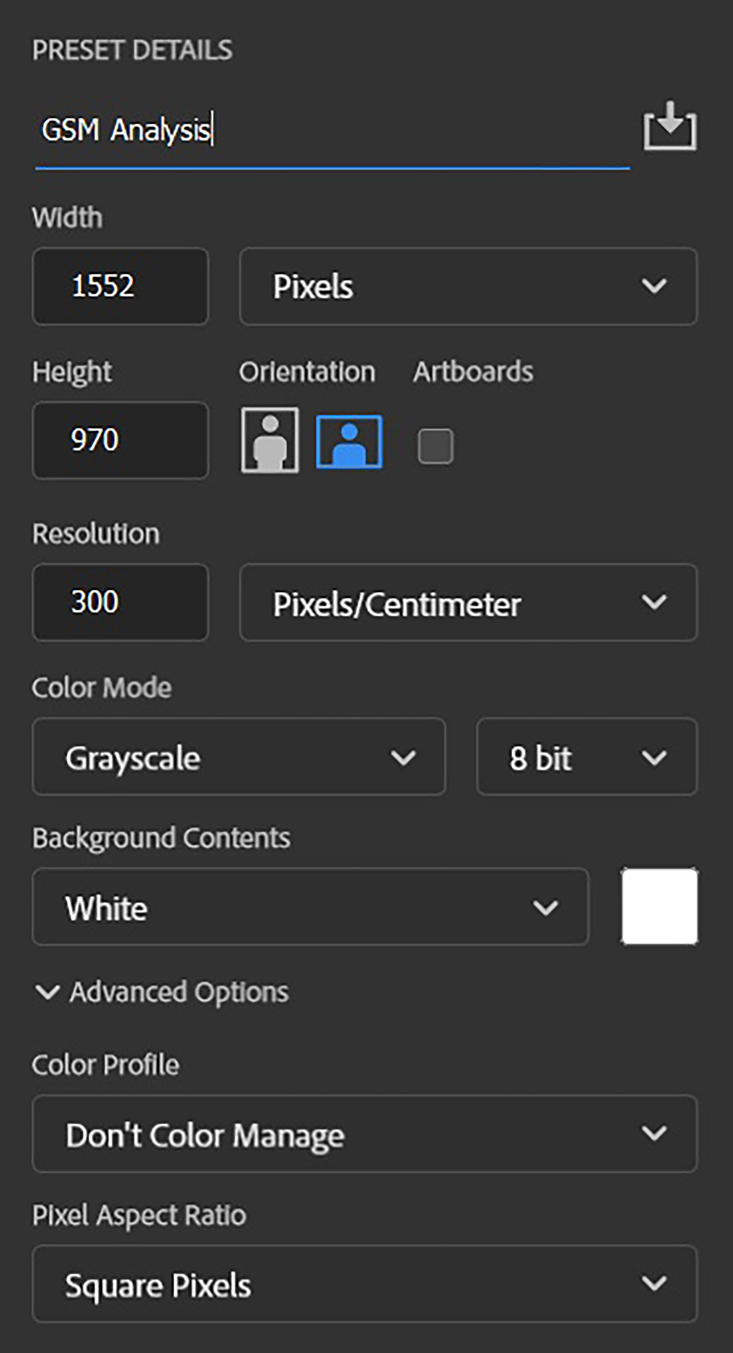
Standardization of image files for grayscale analysis, with 300 dpi resolution and 8-bit grayscale color mode. *Pathway: File > New.*

Post-processing was performed using the paid subscription version of Photoshop 23.1.1 (Adobe, Mountain View, California, United States), in common with the majority of studies that perform GSM analysis by post-processing.^10^^-^ 13 The image files were converted to 8-bit, 300 dpi grayscale format (Figure 1) before adjustment of curves and GSM analysis.

**Figure 2:**
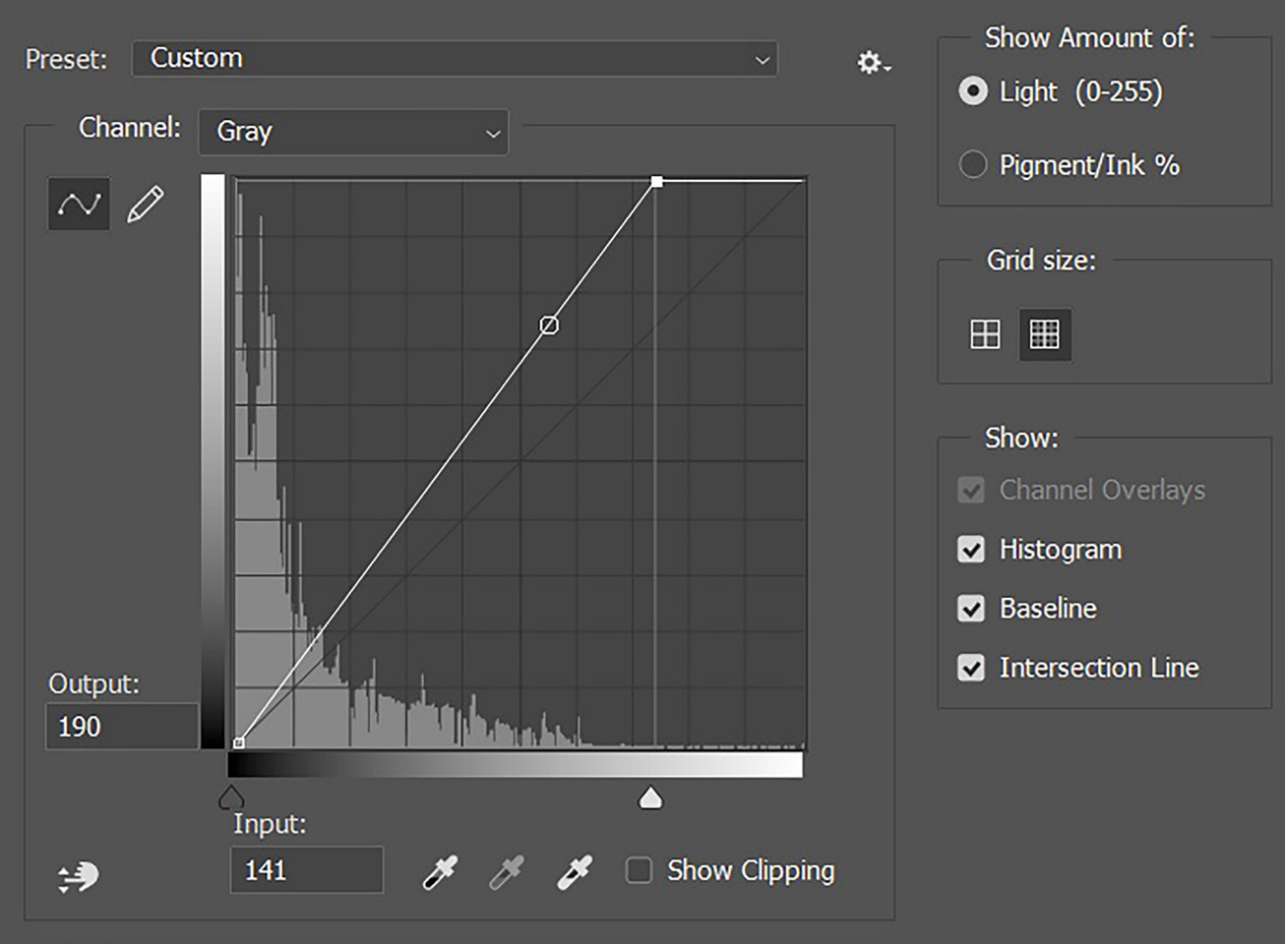
Careful adjustment of the gray channel curves, showing the color of the adventitia in the acquired image at 141, adjusted to 190, which affects the entire image histogram. *Pathway: Image > Adjustments> Curves.*

**Figure 3:**
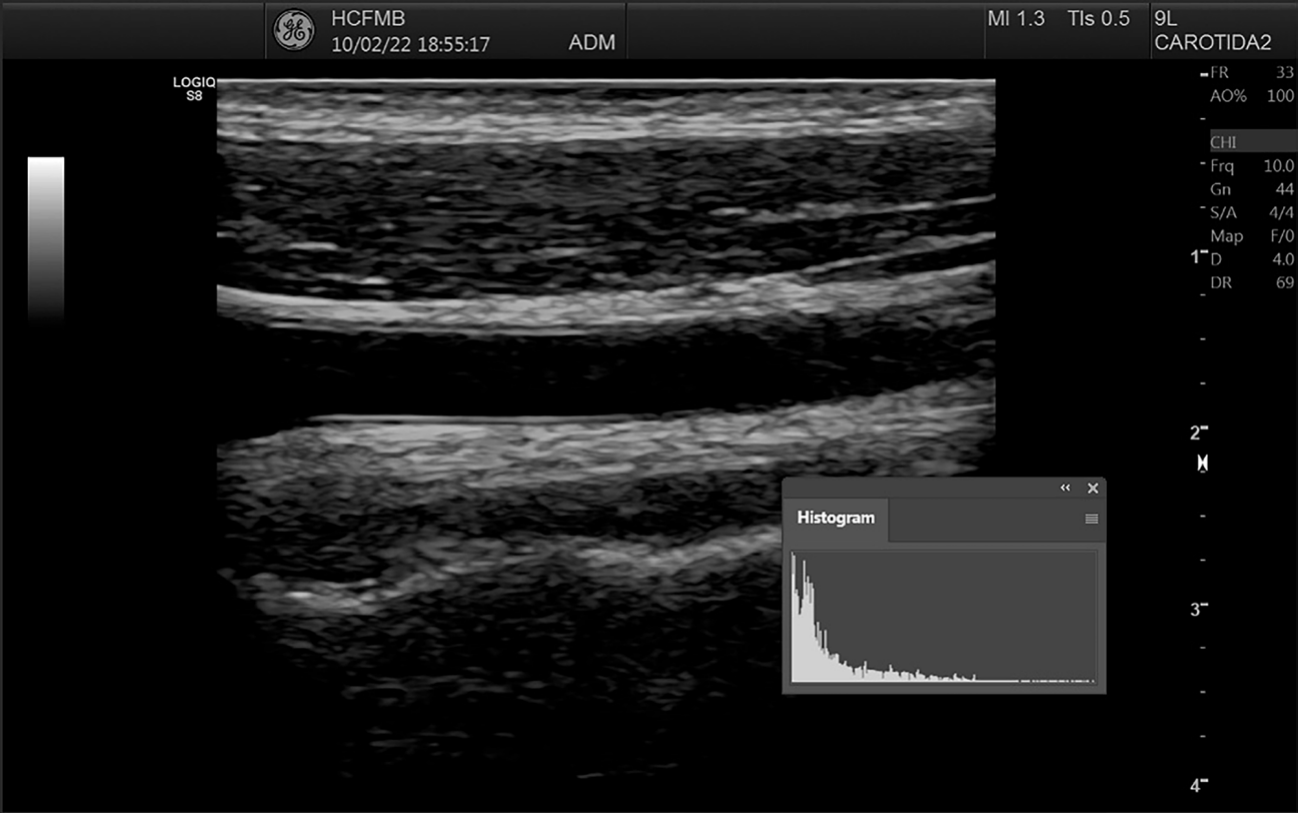
Image and histogram before curve adjustment. *Pathway: Window > Histogram*.

**Figure 4:**
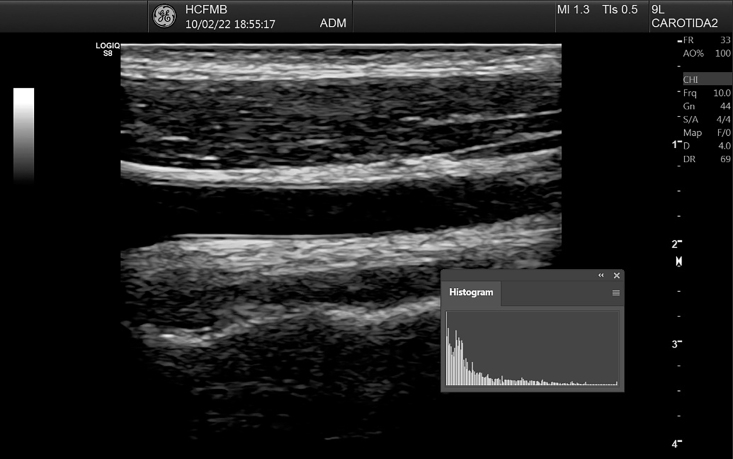
Image and histogram after curve adjustment. *Pathway: Window > Histogram*.

Images were standardized by adjusting the curves of the grayscale histogram. The most widely adopted standardization method involves setting the darkest point of the vascular lumen (blood) to zero and the distal adventitia to 190 (Figure 2).^12^^-^ 16 After this step, it can be observed that the contrast pattern seen previously (Figure 3) changes to a correctly standardized image (Figure 4), maintaining similar histogram curve characteristics. When analyzing GSM, it is essential to remember that each pixel in an 8-bit grayscale image has a value from zero to 255, i.e. 256 different tone options (two raised to the power of eight), where zero equates to black and 255 to white.

**Figure 5:**
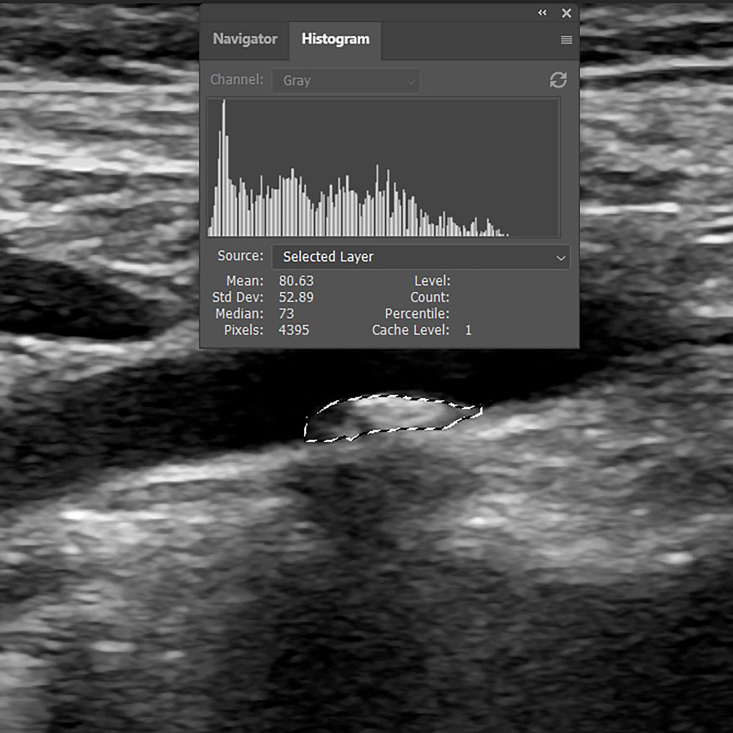
Selection and analysis of the histogram for the region of the atherosclerotic plaque, providing the grayscale median. *Pathway: Window > Histogram*. *Tools > Lasso Tool.*

After standardization, the region of interest is selected and analyzed using the “histogram” tool, which enables the user to obtain the mean, median, and standard deviation of the values of the pixels in a given area (Figure 5). The method used to select the region of interest can be automatic, semiautomatic, or manual. The GSM intervals observed represent different tissues, as shown in Table 1.

**Table 1:** Grayscale median (GSM) values and intervals observed by Lal et al.,^13^ according to the echogenicity of each tissue.

	**GSM**	
	**Median**	**Interval**
**Blood**	2	0-4
**Fat**	12	8-26
**Muscle**	53	41-76
**Fibrotic tissue**	172	112-196
**Calcium**	221	211-255

### Post-processing: posterizing, and color remapping

Although they do not constitute a source of reproducible data (on the contrary, they are a result of the data), posterizing and color remapping enable grayscale images to be enhanced, recoloring different ranges of tones and, in the ultimate analysis, they are useful additional methods for presenting data in an accessible and educational manner.^7^^,^ 13 ^,^ 15 ^,^ 16

**Figure 6:**
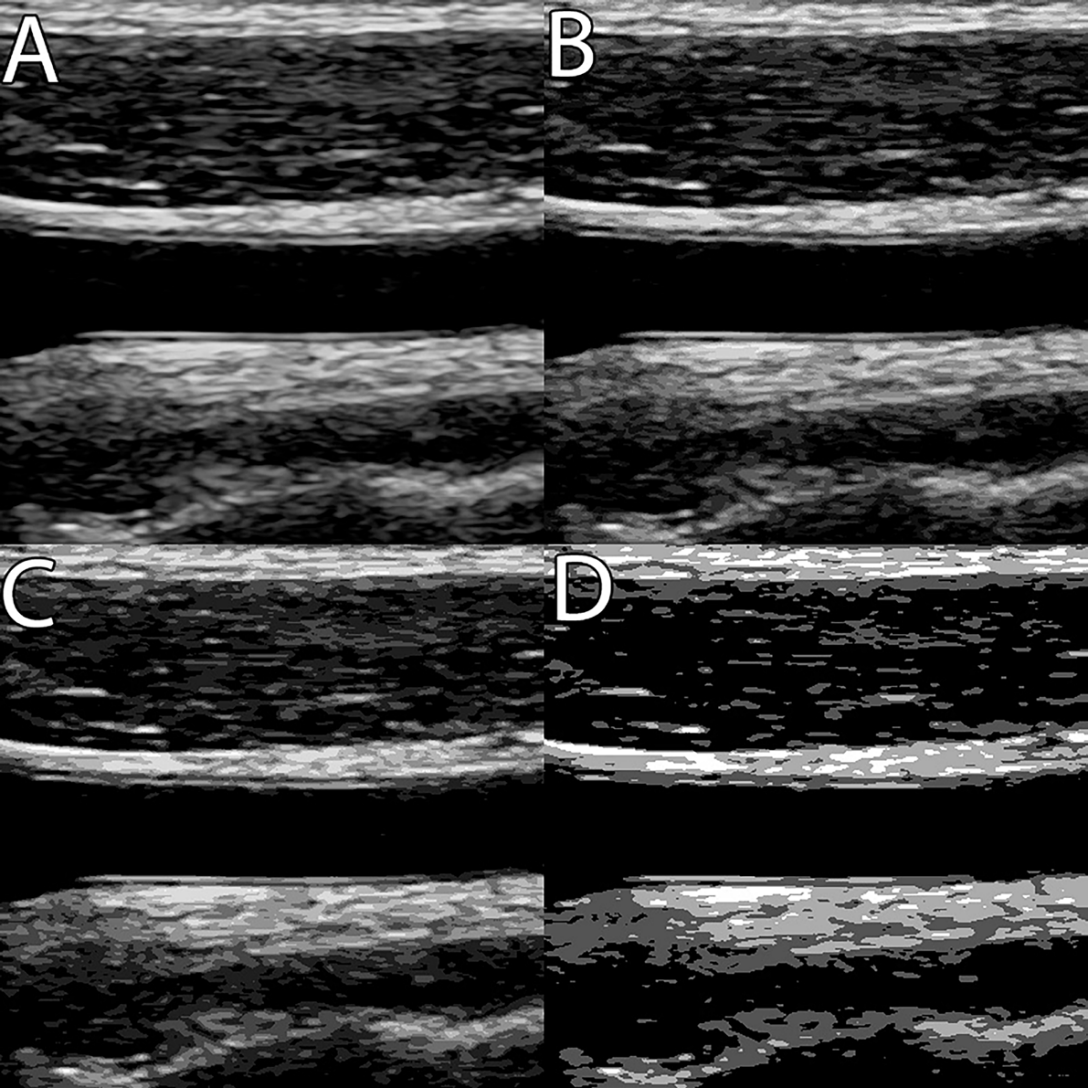
Progressive posterization of an image (A > B > C > D), with reduction of the gray gradient available and simplification of the image. *Pathway: Image > Adjustments > Posterize.*

**Figure 7:**
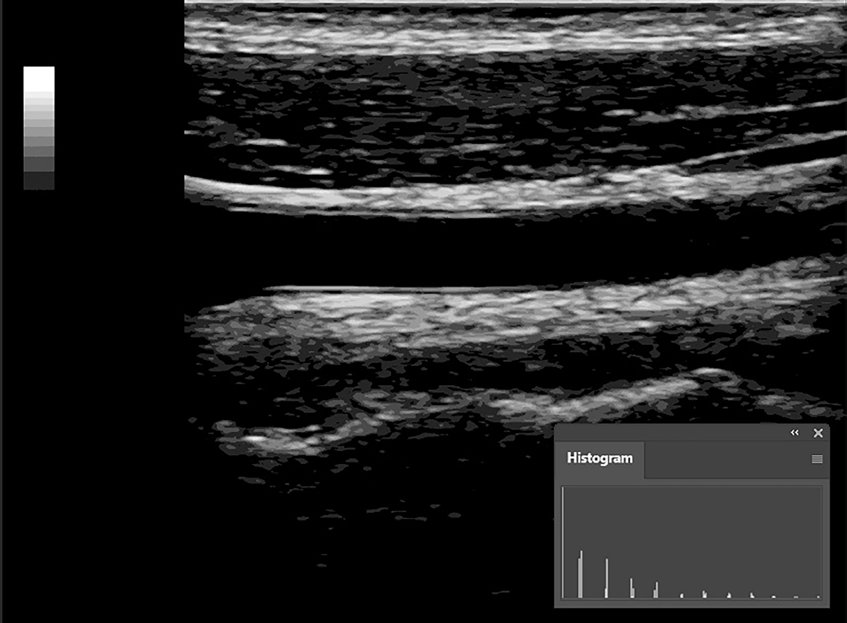
Simplified histogram after posterization of the image. *Pathway: Window > Histogram*.

Posterizing consists of converting an image with a continuous gradation of tones (as mentioned above, 256 different tones for 8-bit grayscale images) into an image with fewer tones, resulting in more abrupt transitions from one tone to the next, which become perceptible to the human eye (Figures 6 and 7). Most image editing programs offer a posterizing function. The gradient of tones can vary depending on how much information the researcher wishes to suppress to create a simplified version of the original grayscale image. We performed posterizing using the same software, following the path: image > adjust > posterize.

**Figure 8:**
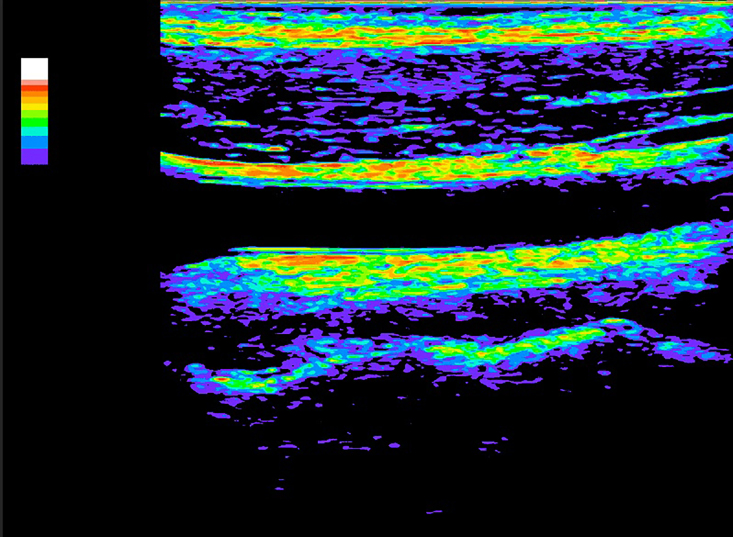
Image resulting from color remapping of a grayscale image.

**Figure 9:**
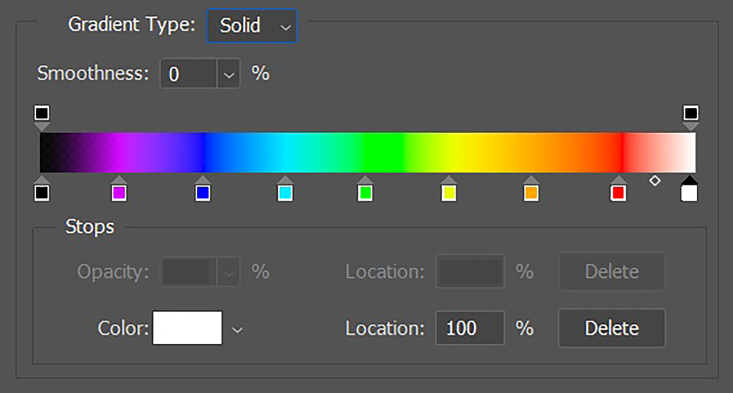
Color gradient used in conversion and remapping of the image. *Pathway: Layer > New Adjustment Layer > Gradient Map.*

Color remapping is conversion of specific ranges of tones to a color gradient, enabling colored images to be created from grayscale images (Figures 8 and 9).

Although color mapping is widely used in all fields of science and radiology, there is no standard color map for analysis of GSM in ultrasound images, since it is not an essential step in the examination. The color map is therefore always subject to the researcher’s personal choice or predetermined by the software of the machine being used. In the present case, we used a classic spectrum of visible colors, from violet to red, with black at the lower limit of the gradient (GSM = 0) and white at the upper limit (GSM > 190). Using this spectrum, blue and purple colors represent GSM < 30 and, therefore, greater risk.

To perform color remapping, we use a layer mask over the standardized and posterized image. Once more, we use the same software as for the previous steps, following the path: layer > new adjustment layer > gradient map.

**Figure 10:**
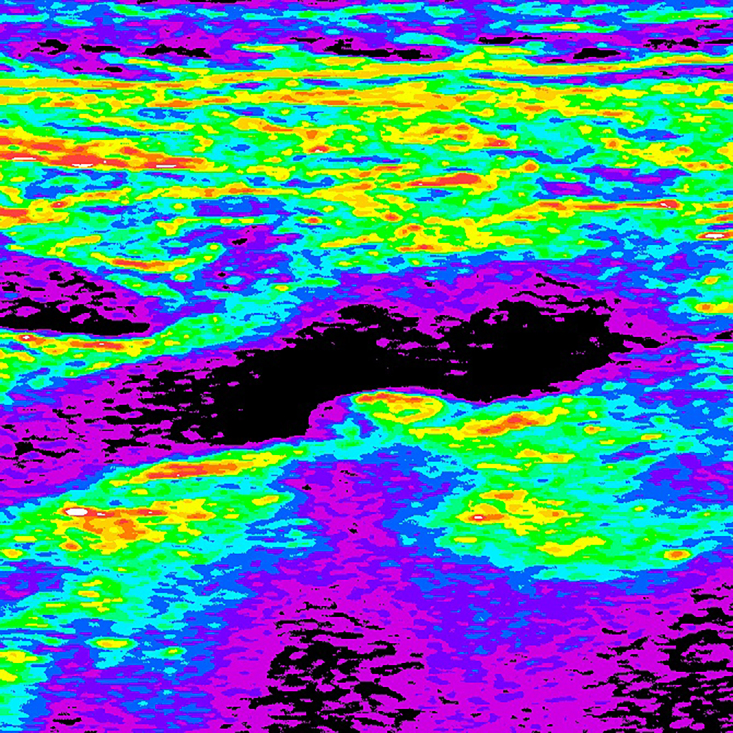
Image resulting from color remapping of a grayscale image.

**Figure 11:**
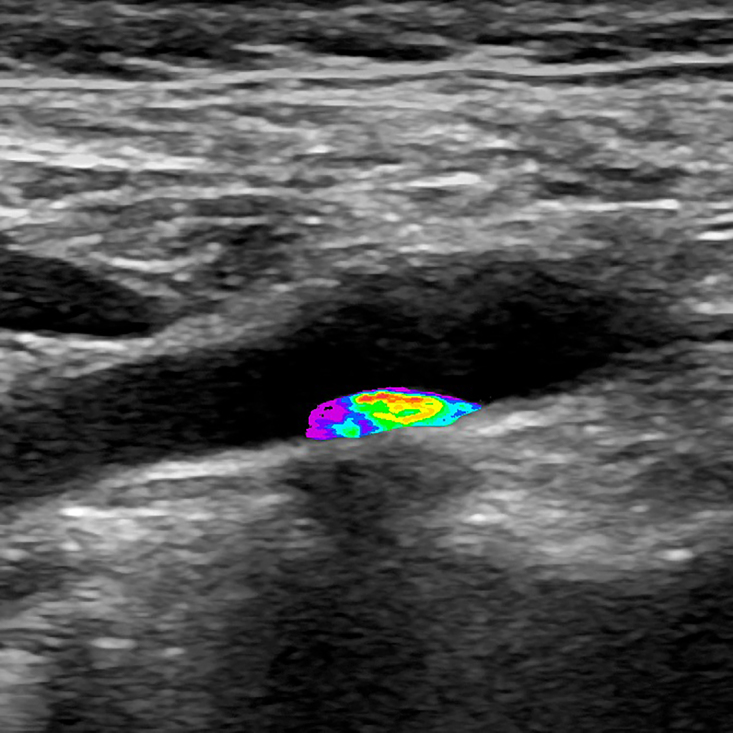
Selection of the region of interest (atherosclerotic plaque) and removal of the color remapping from the remainder of the image. *Pathway: Tools > Lasso Tool. Select inverse, delete.*

After color remapping, it is possible to restrict the color gradient to the region of interest only, for a visually instructive presentation (Figures 10 and 11).

## DISCUSSION

Although there is robust literature supporting the applicability of GSM to identification of patients at high risk of stroke, demonstrating that it is an important instrument for therapeutic decision-making, it is still little used.^14^ It is possible that this is because of the difficulties involved in conducting the analysis, which requires specific software that is not widely available in ultrasound equipment.

As a contribution to dissemination of the technique, assessment using post-processing is a viable alternative, since it only requires image processing software and a computer - which are instruments that are widely available in vascular laboratories and radiology departments.

When compared with other techniques for evaluation of atherosclerotic plaques, GSM offers an important advantage, since it eliminates the subjective component of evaluation. The examination’s objectivity is shown by its high interobserver correlation.^7^^,^ 10 ^,^ 11

Notwithstanding, GSM assessment is still not completely free from subjectivity if a standardized method is not employed, especially considering the extent to which different ultrasound machines and a slight change in B-mode gain can cause large changes in GSM.^15^

To achieve the greatest possible degree of standardization, it is essential to use post-processing by curve editing, rendering the new image after definition of known values, such as blood and the adventitia. Automatic image standardization by software, as is suggested by some authors,^10^^,^ 17 can lead to significant distortions and compromise inter-observer reproducibility and comparisons, and can become even more discrepant if observations are made automatically using different ultrasound machines.^15^

## CONCLUSIONS

We have access to a vast literature on the association between percentage stenosis observed in the extracranial internal carotid and its correlation with risk of stroke. Currently, boosted by developments in minimally invasive or noninvasive diagnostic techniques, most efforts are focused on assessment of the atherosclerotic plaque individually, attempting to identify factors that could contribute to therapeutic decision-making.
