# Supplementary material for: Grayscale median (GSM) post-processing, posterizing, and color mapping for carotid ultrasound
Source: J Vasc Bras. 2023 Feb 10;22:e20220081. doi: 10.1590/1677-5449.202200811 (PMC9925061; doi:10.1590/1677-5449.202200811)
Supplement: Translation

# Translation: Pós-processamento da mediana de escala de cinza (GSM), posterização e remapeamento de cores para ultrassonografia de carótidas

**DOI:** 10.1590/1677-5449.202200812

**Authors:** Pedro Luciano Mellucci, Matheus Bertanha, Rodrigo Gibin Jaldin, Winston Bonetti Yoshida, Marcone Lima Sobreira

**Affiliations:** 1 Universidade Estadual Paulista - UNESP, Botucatu, SP, Brasil.

**Keywords:** doenças das artérias carótidas, estenose das carótidas, ultrassonografia Doppler, pós-imagem, interpretação de imagem assistida por computador

## Abstract

Fatores relativos à placa aterosclerótica podem indicar instabilidade como ulcerações, hemorragias intraplaca, núcleo lipídico, capa fibrosa delgada ou irregular e inflamação. A mediana de escala de cinza (GSM, de *greyscale median*) da placa é um dos métodos mais difundidos de estudo da placa aterosclerótica; nesse sentido, é importante criar uma padronização da pós-processamento de forma compreensível. O pós-processamento foi realizado no *software* Photoshop 23.1.1. A padronização da imagem foi alcançada com o ajuste de curvas do histograma de escalas de cinza definindo o ponto mais escuro do lúmen vascular (sangue) para zero e a adventícia distal para 190. A posterização e o remapeamento de cores foram realizados. Um método que apresenta o atual estado da arte da técnica de forma acessível e ilustrativa pode contribuir para disseminação da análise de GSM. Neste artigo, esse processo é demonstrado passo a passo.

## INTRODUÇÃO

Estudos randomizados realizados entre os anos de 1990 e 2000 identificaram o risco de acidente vascular cerebral (AVC) em pacientes sintomáticos e assintomáticos com doença carotídea extracraniana, baseado no percentual de estenose observado na carótida interna, abrangendo um número considerável de pacientes e determinando diretrizes que são seguidas globalmente para a realização de endarterectomia ou angioplastia carotídea com base no grau de estenose^1^^-^ 5. O principal mecanismo fisiopatológico dos eventos isquêmicos cerebrais decorre da embolização de *debris* ateroembólicos ou de trombos originados a partir de placas instáveis, localizadas principalmente nas bifurcações carotídeas^6^.

O AVC de origem carotídea extracraniana, entretanto, não apresenta a hipoperfusão cerebral como seu principal mecanismo fisiopatológico. Nesse caso, o descolamento de *debris* ateroembólicos e trombos com origem na instabilidade de placas é o principal fator que leva ao AVC^6^.

A utilização do percentual de estenose como único indicativo para a abordagem cirúrgica tem sido apontada como insuficiente, sendo discutida a inclusão de novos marcadores relacionados com as características da placa, como ulcerações, volume do núcleo lipídico, presença de hemorragia intraplaca, espessura de capa fibrosa e inflamação, fortalecendo o conceito de placa instável como determinante para indicação cirúrgica em detrimento da estenose como preditor único. Diversos fatores podem estar associados à instabilidade das placas carotídeas, dando origem a um extenso campo de estudo que busca definir preditores de eventos isquêmicos cerebrais em detrimento do percentual de estenose isoladamente, como ulcerações, hemorragias intraplaca, centro lipídico, capa fibrosa delgada ou irregular e inflamação^7^^,^ 8.

Entre os fatores relativos à placa aterosclerótica mais extensivamente estudados, está a mediana de escala de cinza (GSM, de *greyscale median*) da placa. Em um estudo publicado em 2010 pelo grupo de estudo Asymptomatic Carotid Stenosis and Risk of Stroke (ACSRS), observou-se que valores de GSM inferiores a 30 estariam associados a um risco aumentado de eventos ateroembólicos, com aumento significativo do risco relativo em valores inferiores a 15^7^.

Embora o estudo da GSM tenha sido amplamente difundido na literatura, ele não é realizado rotineiramente pela maior parte dos ultrassonografistas, sendo restrito a poucos centros formadores e de pesquisa, possivelmente porque a maior parte dos *hardwares* (aparelhos) de ultrassonografia não apresenta um *software* (programa) para avaliação automatizada da GSM. Há, ainda, uma grande heterogeneidade de informações acerca do método realizado para alcançar a medida da GSM, desde *softwares built-in* 9 - ou seja, programas instalados no próprio aparelho de ultrassom - até pós-processamento - a avaliação da imagem ecográfica em um computador.

Para contribuir para a difusão da técnica e melhorar a reprodutibilidade, é importante criar uma padronização da pós-análise de forma elucidativa e compreensível ao ecografista sem formação técnica em pós-processamento de imagens.

## MATERIAIS E MÉTODOS

### Aquisição da imagem

Imagens ultrassonográficas em modo B foram obtidas por um único ultrassonografista titulado pelo Colégio Brasileiro de Radiologia e Diagnóstico por Imagem (CBR) e pela Sociedade Brasileira de Angiologia e de Cirurgia Vascular (SBACV), utilizando um único aparelho Logiq S8 (General Electric, Boston, Massachusetts, EUA) com um transdutor linear multifrequencial (8,5-11 MHz) ajustado na frequência de 10 MHz. A distância focal foi definida na túnica adventícia posterior, as alavancas de *time gain compensation* (TGC) foram posicionadas centralmente, e todos os parâmetros de ganho foram padronizados para a configuração do *preset* original do aparelho, incluindo a faixa dinâmica de 69 dB. Todas as imagens foram adquiridas em corte longitudinal.

As imagens obtidas apresentavam 1.552 x 970 pixels, contendo uma área de interesse retangular de 934 x 840 pixels após excluídos títulos, botões de configuração, hora, data e régua de tons de cinza. Optamos por realizar o pós-processamento na imagem inteira (de 1.552 x 970 pixels) para evitar cortes indesejados e para preservar a régua de tons de cinza.

### Pós-processamento: padronização da GSM

O pós-processamento foi realizado no *software* Photoshop 23.1.1 (Adobe, Mountain View, Califórnia, EUA), versão paga por assinatura, em conformidade com a maior parte dos estudos que realizaram a análise de GSM a partir de pós-processamento^10^^-^ 13. Os arquivos de imagem foram convertidos em escala de cinza de 8-bits e 300 dpi (Figura 1) antes do ajuste de curvas e da análise da GSM.

**Figura 1:** Padronização do arquivo para avaliação de escala de cinza, com resolução de 300 dpi e modo de cor em *greyscale* de 8 bits. *Pathway: File > New.*

A padronização da imagem foi alcançada com o ajuste de curvas do histograma de escala de cinza. O método mais recorrente de padronização envolve definir o ponto mais escuro do lúmen vascular (sangue) para zero e a adventícia distal para 190 (Figura 2)^12^^-^ 16. Após esse passo, pode-se observar a mudança no padrão de contraste previamente observado (Figura 3) para uma imagem corretamente padronizada (Figura 4), conservando características similares na curva do histograma. Para a análise de GSM, é fundamental entender que, em imagens em tons de cinza de 8-bits, cada pixel apresenta um valor entre zero e 255 ou 256 opções de tom (dois elevado à oitava potência), em que zero representa o preto e 255, o branco.

**Figura 2:** Minucioso ajuste de curvas do canal cinza demonstrando a cor da adventícia da imagem obtida em 141, com ajuste para 190, o que altera todo o histograma da imagem. *Pathway: Image > Adjustments> Curves.*

**Figura 3:** Imagem e histograma avaliado anteriormente ao ajuste de curvas. *Pathway: Window > Histogram*.

**Figura 4:** Imagem e histograma avaliado posteriormente ao ajuste de curvas. *Pathway: Window > Histogram*.

Após a padronização, a área de interesse é delineada e analisada usando a ferramenta “histograma”, que permite ao usuário obter a média, a mediana e o desvio-padrão dos pixels em determinada área (Figura 5). O método de delineamento da área de interesse pode ser automático, semiautomático ou manual. Os intervalos de GSM observados representam diferentes tecidos, conforme demonstra a Tabela 1.

**Figura 5:** Delimitação e avaliação do histograma da área da placa aterosclerótica, com obtenção da mediana de escala de cinza. *Pathway: Window > Histogram*. *Tools > Lasso Tool.*

**Tabela 1:** Valores de mediana de escala de cinza (GSM, de *greyscale median*) e intervalos observados por Lal et al.^13^, de acordo com a ecogenicidade do tecido estudado.

	GSM	
	Mediana	Intervalo
**Sangue**	2	0-4
**Gordura**	12	8-26
**Músculo**	53	41-76
**Tecido fibrótico**	172	112-196
**Cálcio**	221	211-255

### Pós-processamento: posterização e remapeamento de cores

Embora não seja fonte de dados reprodutível (ao contrário, sendo um resultado dela), a posterização e o remapeamento de cores permite o aprimoramento de imagens em tons de cinza e a recolorização de diferentes faixas de tons, sendo, em última análise, usados como uma forma adicional de apresentação de dados de forma acessível e educacional^7^^,^ 13 ^,^ 15 ^,^ 16.

A posterização é a conversão de uma imagem com uma graduação contínua de tons (conforme mencionado, 256 tons para imagens em tons de cinza de 8-bits) em uma imagem com menos tons, resultando em mudanças mais abruptas de um tom para o outro, que são perceptíveis ao olho humano (Figuras 6 e 7). A maior parte dos *softwares* de edição de imagem apresenta funcionalidades para posterização. O gradiente desses tons pode variar de acordo com a quantidade de informação que o pesquisador deseja suprimir para criar uma versão simplificada da imagem original em cinza. Para realizar a posterização, utilizamos o mesmo *software*, seguindo o caminho: image > adjust > posterize.

**Figura 6:** Posterização progressiva de uma imagem (A > B > C > D), com redução do gradiente de cinza disponível e simplificação da imagem. *Pathway: Image > Adjustments > Posterize.*

**Figura 7:** Histograma simplificado após posterização da imagem. *Pathway: Window > Histogram*.

O remapeamento de cores é a conversão de uma determinada faixa de tons em um gradiente de cores, permitindo, portanto, a criação de uma imagem colorida a partir de uma imagem em tons de cinza (Figuras 8 e 9).

**Figura 8:** Imagem resultante de um remapeamento de cores de uma imagem em tons de cinza.

**Figura 9:** Gradiente de cores utilizado na conversão e remapeamento da imagem. *Pathway: Layer > New Adjustment Layer > Gradient Map.*

Embora amplamente utilizado em todos os campos da ciência e radiologia, não existe um mapa de cores padronizado para a avaliação de GSM no ultrassom, não sendo um passo essencial para a realização do exame. Dessa forma, o mapa de cores é constantemente sujeito à escolha pessoal do pesquisador ou predeterminado pelo *software* do aparelho utilizado. Nesse caso, utilizamos o espectro clássico de cor visível, do violeta ao vermelho, com a cor preta no limite inferior do gradiente (GSM = 0) e a cor branca no limite superior (GSM > 190). Nesse espectro, especificamente, cores azuis e roxas representam GSM < 30 e, portanto, maior risco.

Para realizar o remapeamento de cores, utilizamos uma máscara de *layer* sobre a imagem padronizada e posterizada. Novamente, utilizamos o mesmo *software*, seguindo o caminho: layer > new adjustment layer > gradient map.

Posteriormente ao remapeamento de cores, é possível definir apenas a área de interesse para uma apresentação visualmente didática (Figuras 10 e 11).

**Figura 10:** Imagem resultante de um remapeamento de cores de uma imagem em tons de cinza.

**Figura 11:** Delimitação de área de interesse (placa aterosclerótica) e exclusão do remapeamento de cores do restante da imagem. *Pathway: Tools > Lasso Tool. Select inverse, delete.*

## DISCUSSÃO

Embora a medida da GSM apresente uma literatura robusta que defenda a sua aplicabilidade para identificação de pacientes com um alto risco para desenvolvimento de AVC, servindo como importante instrumento na decisão terapêutica, a sua difusão ainda é restrita^14^. É possível que isso se deva à dificuldade de realização do exame, que depende de um *software* específico que não está amplamente disponível nos aparelhos de ultrassonografia.

Para contribuir para a disseminação da técnica, a avaliação a partir de pós-processamento apresenta-se como uma alternativa viável, pois depende apenas de um *software* para processamento de imagens e de um computador - instrumentos amplamente disponíveis nos laboratórios vasculares e departamentos de radiologia.

Quando comparada com as demais técnicas para avaliação da placa aterosclerótica, a GSM apresenta importante superioridade por afastar o componente subjetivo da avaliação. A objetividade do exame é constatada a partir de uma alta correlação interobservadores^7^^,^ 10 ^,^ 11.

A avaliação a partir da GSM não é, entretanto, completamente isenta de subjetividade se um método padronizado não for aplicado, especialmente se considerando a medida em aparelhos de ultrassonografia distintos e que uma breve alteração no ganho do modo B pode acarretar uma grande mudança da GSM^15^.

Para alcançar o maior nível possível de padronização, o pós-processamento a partir da edição das curvas, com renderização de nova imagem após definir valores conhecidos, como o sangue e a adventícia, é fundamental. A padronização automática da imagem pelo próprio *software* conforme postulado por alguns autores^10^^,^ 17 pode levar a distorções significativas e prejudicar a reprodutibilidade e comparação entre observadores, que pode ser ainda mais discrepante se a observação for realizada automaticamente entre aparelhos distintos^15^.

## CONCLUSÃO

Dispomos de uma vasta literatura acerca da associação entre o percentual de estenose observado na carótida interna extracraniana e sua correlação com o risco de AVC. Atualmente, alavancada pela evolução das técnicas diagnósticas minimamente invasivas ou não invasivas, a maior parte dos esforços está concentrada na avaliação da placa aterosclerótica individualmente, buscando identificar fatores que possam contribuir na decisão terapêutica.
